# Brain Derived Neurotrophic Factor and Glial Cell Line-Derived Neurotrophic Factor-Transfected Bone Mesenchymal Stem Cells for the Repair of Periphery Nerve Injury

**DOI:** 10.3389/fbioe.2020.00874

**Published:** 2020-07-30

**Authors:** Qiang Zhang, Ping Wu, Feixiang Chen, Yanan Zhao, Yinping Li, Xiaohua He, Céline Huselstein, Qifa Ye, Zan Tong, Yun Chen

**Affiliations:** ^1^Department of Biomedical Engineering and Hubei Province Key Laboratory of Allergy and Immune Related Diseases, School of Basic Medical Sciences, Wuhan University, Wuhan, China; ^2^Hangzhou Singclean Medical Products Co., Ltd., Hangzhou, China; ^3^CNRS UMR 7561 and FR CNRS-INSERM 32.09, Nancy University, Vandæuvre-lès-Nancy, France; ^4^Zhongnan Hospital of Wuhan University, Institute of Hepatobiliary Diseases of Wuhan University, Transplant Center of Wuhan University, Wuhan, China; ^5^Hubei Engineering Center of Natural Polymers-Based Medical Materials, Wuhan University, Wuhan, China

**Keywords:** poly (L-lactic acid), soy protein isolate, bone marrow mesenchymal stem cells, brain derived neurotrophic factor, glial cell line-derived neurotrophic factor

## Abstract

Peripheral nerve injury is a common clinical neurological disease. In our previous study, highly oriented poly (L-lactic acid) (PLLA)/soy protein isolate (SPI) nanofiber nerve conduits were constructed and exhibited a certain repair capacity for peripheral nerve injury. In order to further improve their nerve repairing efficiency, the bone mesenchymal stem cells (BMSCs) overexpressing brain derived neurotrophic factor (BDNF) and glial cell line-derived neurotrophic factor (GDNF) were introduced into the conduits as seed cells and then were used to repair the 10-mm sciatic nerve defects in rats. The nerve repair efficiency of the functional nerve conduits was evaluated by gait experiment, electrophysiological test, and a series of assays such as hemotoxylin-eosin (HE) staining, immunofluorescence staining, toluidine blue (TB) staining, transmission electron microscopy (TEM) observation of regenerated nerve and Masson’s trichrome staining of gastrocnemius muscle. The results showed that the conduits containing BMSCs overexpressing BDNF and GDNF double-factors group had better nerve repairing efficiency than blank BMSCs and single BDNF or GDNF factor groups, and superior to autografts group in some aspects. These data demonstrated that BDNF and GDNF produced by BMSCs could synergistically promote peripheral nerve repair. This study shed a new light on the conduits and stem cells-based peripheral nerve repair.

## Introduction

Peripheral nerve injury (PNI) is a critical issue in the field of regenerative medicine (Wang et al., 2018; Li et al., 2020). Accelerating axonal regeneration and improving functional recovery after PNI is a clinical dilemma and a basic medical challenge (Li et al., 2019; Sayad-Fathi et al., 2019). In clinical practice, end-to-end suturing of proximal and distal stumps is the ideal method to repair short nerve defect (Guo et al., 2019; Wu et al., 2019). The gold standard of long nerve regeneration is autograft (Yi et al., 2018; Vijayavenkataraman, 2020). However, autograft requires sacrifice of a functional nerve, which may result in donor nerve sensory loss and neuropathic pain (Guo et al., 2018; Wu et al., 2020). Hence, the use of nerve guide conduits could avoid these problems (Chrzaszcz et al., 2018; Chen et al., 2019; Vijayavenkataraman et al., 2019). However, the reported nerve conduits could not fully meet the demands for quick and effective nerve repair (Carvalho et al., 2019; Riccio et al., 2019).

The classical strategy of tissue engineering is to construct composite nerve conduits with biodegradable polymer materials combined with seed cells or neurotrophic factors (Zhang et al., 2019). The addition of neurotrophic factors in nerve conduit could significantly improve the efficiency of nerve regeneration (Gao et al., 2016). Directly introducing exogenous neurotrophic factor into nerve guide conduit was reported (Labroo et al., 2018; Lin et al., 2019). However, these exogenous neurotrophic factors in the nerve conduits are easy to be lost or become inactive (Hobson et al., 2000; Moskow et al., 2019). What’s worse, excessive exogenous neurotrophic factors may cause trapping of regenerating axons and formation of nerve coils (Eggers et al., 2013).

Therefore, seed cells producing neurotrophic factors were widely used to overcome these problems (Hsueh et al., 2014; Zhu et al., 2014; Hsu et al., 2017). Bone marrow mesenchymal stem cells (BMSCs) are most promising seed cells for nerve repair and regeneration (Gao et al., 2016; Xue et al., 2017). BMSCs had lots of advantages including wide range of sources, easy to isolate and culture, and immunological naivety (Cho et al., 2018; Cui et al., 2018). BMSCs could suppress neuronal cell death and promote nerve regeneration in conduit guided sciatic nerve repair in rats (Hsu et al., 2013). BMSCs producing BDNF were reported to promote motor functional recovery in spinal cord transfected rat (Xiong et al., 2016).

However, single neurotrophic factor is often not very effective (Li et al., 2006; Cangellaris and Gillette, 2018). Various endogenous neurotrophic factors for nerve regeneration were reported (Tajdaran et al., 2018; Liu et al., 2020). Among them, BDNF can promote the myelinization of neogenesis nerve (Lopes et al., 2017), GDNF can protect motor neurons from injury caused by nerve transection, and improve the re-innervation function of nerves (Eggers et al., 2008). Many studies have confirmed the role of BDNF and GDNF in PNI, but their synergy at a ratio of 1:1 has not been studied *in vivo* (Zurn et al., 1996; Fu et al., 2011; Hoyng et al., 2014; Wang et al., 2016; Hsu et al., 2019).

Our previous work has shown that PLLA/SPI composite nanofiber conduits (HO-PSNCs) can promote nerve regeneration. The nerve conduits were modified with biochemical cues by SPI blending and topographical cues by highly oriented electrospinning. The two strategies combined together could improve the hydrophilicity and biodegradability of the biomaterials, and promote neural cell growth, spreading, extension, and neurite outgrowth *in vitro*, and support the nerve regeneration *in vivo* (Zhang et al., 2020). In order to further elevate the efficiency of peripheral nerve repair, we constructed BDNF transfected BMSCs and GDNF transfected BMSCs, and then the BMSCs were introduced into HO-PSNCs conduits to bridge sciatic nerve defects in rats. Two factors system were compared to single factor system, which provide evidence for the synergistically application of endogenous neurotrophic factors in nerve regeneration.

## Experimental Section

### Materials

PLLA with a molecular weight of 150 kDa was supplied by Shenzhen Polymtek Biomaterial Co., Ltd (Shenzhen, China). Soy protein isolate (SPI) with weight-average molecular weight (M_w_) of 2.05 × 10^5^ was purchased from DuPont Protein Technology (Luohe, China). Other chemicals were of analytical grade agents.

### Preparation of the HO-PSNCs Scaffolds

The HO-PSNCs nerve conduits (Highly oriented PLLA/SPI nanofibrous conduits) were prepared as previous work (Zhang et al., 2020). In brief, 10 g PLLA was added to 90 g hexafluoropropanol. Two gram SPI powder was added to 98 g hexafluoroisopropanol. PLLA solution and SPI solution were mixed at weight ratio of 80:20. The PLLA/SPI composite solution was added into a 5 mL syringe with a needle for electrospinning. The prepared nanofiber conduits were stored in a dryer.

### BMSCs Culture and Identification

The BMSCs were isolated from the adult Sprague Dawley rats (120∼150 g). After the rats were euthanized, the rat femurs were dissected out and the marrow cavities were exposed. The marrow cavities were washed with α-modified Eagle’s medium (α-MEM, Gibco) to collect the BMSCs. After the 1000 rpm centrifugal precipitation, the cells were re-suspended with complete α-MEM [containing 10% fetal bovine serum (FBS) and 1% penicillin-streptomycin (Gibco)]. Then the cells were cultured in a T75 flask at 37°C with 5% CO_2_. After 72 h, the medium was replaced with fresh complete α-MEM. The cells were confirmed as BMSCs by the flow cytometry of evaluating the expression of CD11 and CD45, while the CD29 and CD90 were the negative control. Optical images and SEM images were also taken to exam the BMSCs.

### Lentivirus Construction

The Trizol reagent (Invitrogen, United States) was used for RNA extraction. The reverse transcription was then performed with the cDNA Reverse Transcription Kit (Bio-Rad, United States). Lentiviral vectors pCDH-CMV-MCS-EF1-copGFP-T2A-Puro-BDNF and pCDH-CMV-MCS-EF1-copGFP-T2A-Puro-GDNF vectors were constructed using PCR. The primer sequences were as follows: BDNF, 5′-GCG GGA TCC GCC ACC ATG GTG ACC ATC CTT TTC CTT AC-3′ and 5′-GCG GCG GCC GCC TAT CTT CCC CTT TTA ATG G-3′; GDNF, 5′-GCG GGA TCC GCC ATT ATG GGA TGT CGT GGC TG-3′ and 5′-GCG GCG GCC GCT CAG ATA CAT CCA CAC CGT TTA GC-3′. The lentiviral packaging vectors (pLP1, pLP2, pLP) were co-transfected along with pCDH-CMV-MCS-EF1-copGFP-T2A-Puro, pCDH-CMV-MCS-EF1-copGFP-T2A-Puro-BDNF, or pCDH-CMV-MCS-EF1-copGFP-T2A-Puro-GDNF into 293T cells using Lipofectamine 2000 (Invitrogen, United States). After 48 h of transfection, the lentiviruses were collected after filtering the supernatant of cell culture medium.

### Western Blot Analysis

Lentivirus overexpressing BDNF and GDNF were transfected into BMSCs for 48 h, and the overexpression of BDNF and GDNF in BMSCs were identified by western blot. The BMSCs (P3) were harvested and washed with cold PBS, then incubated with primary antibodies: anti-BDNF antibody (A16299, Abclonal, China, 1:1000), anti-GDNF antibody (A14639, Abclonal, China, 1:1000) and anti-β-actin antibody (GB1101, Servicebio, China, 1:2000). HRP signals were detected by Image Studio Digits Ver 4.0. Density values were normalized to β-actin and results are representative of three independent experiments.

### Cell Seeding

The conduits were cut into 12 mm, washed with PBS twice, and soaked in 75% alcohol for 72 h and then repeatedly washed three times with PBS. The alcohol-sterile conduits were seeded with BMSCs overexpressing GFP (BMSC-Vector group), BMSCs overexpressing BDNF (BMSC-BDNF group), BMSCs overexpressing GDNF (BMSC-GDNF group), and BMSCs overexpressing BDNF and GDNF [BMSC-(BDNF + GDNF) group], respectively. Cell density was 5 × 10^4^/conduit (according to our preliminary experiment), 37°C and 5% CO_2_ under the condition of cultivation for 24 h. The BMSC-(BDNF + GDNF) group is 1:1 combination of BMSC-BDNF and BMSC-GDNF groups. To make the BMSCs distributed evenly, we firstly inoculated 2.5 × 10^4^ cells in the inner wall of the conduits on the one side. We flipped the conduits for 180 degrees after the BMSCs were attached to the conduits for 12 h, then the other 2.5 × 10^4^ BMSCs were inoculated in the inner wall of the conduits on the other side.

### Animals Surgery

All the animals experiment procedures were approved by the Animal Care and Use Committees of Wuhan University and carried out in accordance with the “Guidelines and Regulations for the use and care of Animals of the Review Board of Hubei Medical Laboratory Animal Center”. Adult SD rats (180∼200 g) were used to exam the nerve regeneration performance *in vivo*. The skins of anesthetized rats were cut to expose the right sciatic nerve. Blunt dissection is used to separate the muscles surrounding the nerve tissue. The sciatic nerve was then was severed into proximal and distal segments with 10 mm defects at the center of the right posterior limb. As shown in Table 1, 60 rats were divided into six groups randomly: defects connected with 10 mm autologous nerve grafts (Autograft group), 12 mm HO-PSNCs conduits (Control group), 12 mm HO-PSNCs + BMSCs overexpressing GFP conduits (BMSC-vector group), 12 mm HO-PSNCs + BMSCs overexpressing BDNF conduits (BMSC-BDNF group), 12 mm HO-PSNCs + BMSCs overexpressing GDNF conduits (BMSC-GDNF group), 12 mm HO-PSNCs + BMSCs overexpressing BDNF + BMSCs overexpressing GDNF conduits [BMSC-(BDNF + GDNF) group]. The 8-0 nylon was used to suture the proximal and the distal stumps nerve with depth of 1 mm into the conduits. 6-0 nylon was used to re-suture the muscle and skin layers.

**TABLE 1:** Group codes in animal studies.

Group	12 mm HO-PSNCs	BMSCs overexpressing GFP	BMSCs overexpressing BDNF	BMSCs overexpressing GDNF
Autograft	×	×	×	×
Control	√	×	×	×
BMSC-vector	√	√	×	×
BMSC-BDNF	√	×	√	×
BMSC-GDNF	√	×	×	√
BMSC-(BDNF + GDNF)	√	×	√	√

*The “√” indicates that the actional element was included, and the “×” indicates that the actional element is not included.*

### General Observation

Three months after surgery, SD rats were placed in a clean table and observed the movement behavior in the free environment. The whole movement process and foot condition of the rats were recorded with camera. After observation, SD rats were put into a beaker to observe the recovery of the legs and feet of the surgical side when the rats were standing.

### Walking Track Analysis

In order to assess the behavior of the rats at 3 months after surgery, walking track analysis was performed. In briefly, the rats were walking along with a wooden walking alley. The white papers were put on the floor of the alley. The red paint was applied to the rat’s plantar surface prior before walking on the floor of the alley. As the rats walking along the track, their left and right posterior limb footprints on the track were recorded. The following information was obtained from the footprints: distance from the heel to the top of the third toe (print length; PL); distance between the first and the fifth toe (toe spread; TS) and distance from the second to the fourth toe (intermediary toe spread; IT). These measures were also collected from the non-operated rat posterior limb (information for these posterior limbs are marked as NPL, NTS, and NIT) and the operated, experimental posterior limb (information for these posterior limbs are marked as EPL, ETS, and EIT). In the control groups, information of the right posterior limb was compared with those from the left ones. To calculate the sciatic function index (SFI), the information was fed into the following equation (1) from previous studies:

(1) SFI=-38.3×(EPL-NPL)/NPL+109.5×(ETS-NTS)/NTS+13.3×(EIT-NIT)/NIT-8.8

Interpolating identical values of PL, TS, and IT from the right and the left hind feet results in a value close to zero in normal rats. A value of −100 implies total impairment.

### Electrophysiology Evaluation

Three months after surgery, electrophysiology experiment was performed under anesthesia. The surgical sites were re-opened to expose the sciatic nerve. The electromyography was evaluated by an electrophysiology system (RM6240, China). The 10 mV electrical stimuli were applied to the nerve trunk at the proximal ends of the graft. Compound muscle action potentials (CMAPs) were recorded on the gastrocnemius muscle. The ratios of CMAPs in each group were used to assess the sciatic nerve functional recovery.

### HE Staining and Immunofluorescence Staining

Three months after surgery, the regenerated nerves were harvested and then fixed in 4% paraformaldehyde solution for 48 h. The fixed nerve samples were dehydrated, paraffin-embedded and sectioned into 6-μm thick. A part of sections was stained with HE dying solution, and then observed under a light microscope (TE2000-U, Nikon, Japan). Another part of sections was used for immunofluorescence staining. In brief, the sections were incubated with mouse anti-NF200 antibody (diluted 1:200) and goat anti S100 antibody (diluted 1:50). After thorough washing with PBS, the sections were incubated with fluorescent secondary antibodies (Alexa Fluor-488 or -555 conjugated goat anti-mouse IgG diluted 1:200). Finally, sections were stained by DAPI and observed under a fluorescence microscope (TE2000-U, Nikon, Japan). To quantify the percentages of positive NF200 and S100 staining, fifteen fields of five images were randomly captured at 400 × magnification. The Image-Pro Plus software was used to calculate the percentages of positive NF200 and S100 staining.

### Myelination Analysis

Three months after surgery, the regenerated nerves were collected and fixed in 2.5% glutaraldehyde solution at 4°C for 48 h. The regenerated nerves were then immobilized in 1% osmium acid, dehydrated, embedded and then sliced into 1 μm thick semi-thin sections. Through the toluidine blue staining, the sections were observed by optical microscope. Three sections per sample were randomly chosen and six images per section were randomly taken at 20 × magnification. Then 50 nm ultra-thin sections were stained with lead citrate and uranyl acetate. The ultrastructure of regenerated nerve fibers was observed by transmission electron microscope (TEM, HT7700, Hitachi, Japan). Five images were randomly captured at 400 × magnification and all axons from five images were analyzed. Toluidine blue stained images and TEM images were measured by Image-Pro Plus software to analyze the diameter and density of myelinated nerve fibers, the area of the myelinated axons and myelin sheath thickness.

### Assessment of Gastrocnemius Muscles

Three months after surgery, normal and operative gastrocnemius muscles of the rats were harvested completely, washed with PBS, drained with filter paper, weighed and photographed. According to the equation (2), the muscle weight recovery rate of gastrocnemius was calculated as follows:

(2) Wr(%)=[Ws/Wn]×100

Wr: Muscle weight recovery rate; Ws: muscle weight of gastrocnemius on the operative side; Wn: muscle weight of normal lateral gastrocnemius muscle.

The gastrocnemius tissue was then fixed in 4% paraformaldehyde for more than 24 h. After dehydration, paraffin embedding and sectioning, 6 μm thick paraffin sections were prepared. Then Masson staining was carried out. Three different fields of vision were collected for each section. Image-pro plus software was used to calculate muscle fiber area (a) and collagen fiber area (b). Then, the percentage of collagen fiber area was calculated according to the equation (3):

(3) c(%)=[b/(a+b)]×100

a, cross-sectional area of muscle fibers; b, collagen fiber area; c, percentage of collagen fibers.

### Statistical Analysis

All quantitative data was expressed as mean ± SEM. One-way analysis of variance (ANOVA) followed by *post hoc* test was used for statistics analysis. The difference (*P* < 0.05) was considered to be statistically significant.

## Results

### Identification and Morphology of BMSCs

The light microscope and scanning electron microscope images of BMSCs are shown in Supplementary Figures S1A,B, respectively. The BMSCs were closely clustered, with long fusiform or flat cells and small cell bodies, but the protrusions were whirlpool or radial. The surface markers CD11b, CD45, CD29, and CD90 of BMSCs were identified by flow cytometry, and the results are shown in Supplementary Figures S1C–F. Normally, BMSCs expressed CD29 and CD90 on the cell surface, but not CD11b and CD45 (Zhang et al., 2012). According to Supplementary Figures S1C–F, strong positive CD29 and CD90 signals could be detected in BMSCs, while only weak positive CD11b and CD45 signals could be detected in BMSCs. According to the analysis, the positive expression of CD29 was more than 99%, the positive expression of CD90 was more than 95%, while the positive rate of CD11b and CD45 was less than 1%, indicating that the purity of BMSCs was more than 95%.

### Construction of BDNF and GDNF Transfected BMSCs

**FIGURE 1:**
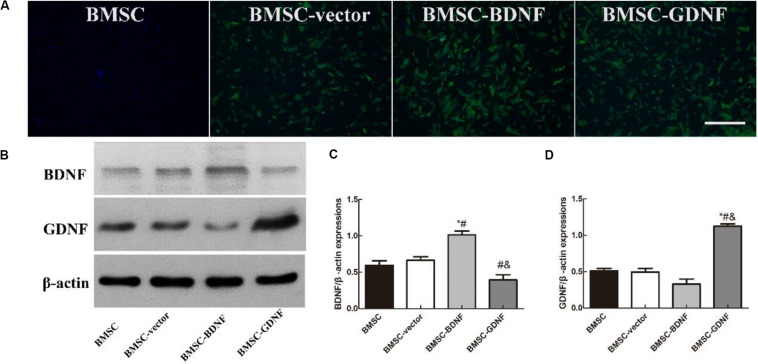
Characterization of BMSC-BDNF and BMSC-GDNF. **(A)** Fluorescence images of BMSCs and BMSCs transfected with blank vectors, BDNF and GDNF. Scale bar = 20 μm. **(B)** The western blot analysis of BDNF and GDNF proteins. **(C)** The BDNF protein levels and **(D)** the GDNF protein levels in the BMSC, BMSC-vector, BMSC-BDNF and BMSC-GDNF groups. *P < 0.05, compared with BMSC group; ^#^*P* < 0.05, compared with BMSC-vector group; ^&^*P* < 0.05, compared with BMSC-BDNF group.

The fluorescence images of BMSCs and BMSCs transfected with blank vectors, BDNF, GDNF are shown in Figure 1A. The BMSCs exhibited green fluorescence in the BMSC-vector, BMSC-BDNF and BMSC-GDNF groups, which could demonstrate the presence of the GFP protein in the three groups. The overexpressing BDNF and GDNF protein were detected by western blot. As shown in Figures 1B,C, higher protein expression levels of BDNF were detected in the BMSC-BDNF group, revealing that BMSCs transfected with BDNF overexpressing lentivirus could effectively express BDNF proteins. In the Figures 1B,D, the GDNF protein in the BMSC-GDNF group was the highest among the four groups, revealing that BMSCs transfected with GDNF overexpressing lentivirus could effectively express GDNF proteins.

### General Observation

**FIGURE 2:**
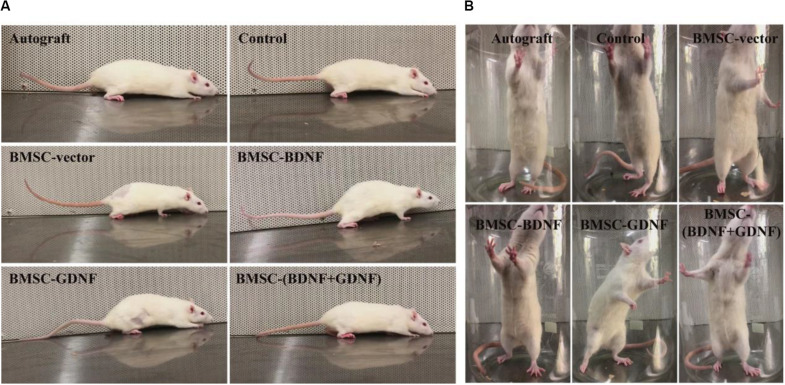
General observation of the movement of rats at 3 months after surgery. Images of walking **(A)** and standing **(B)** of rats in each group.

Three months after surgery, the walking and standing behaviors of the rats were observed, and the images are shown in Figure 2. Compared with the normal posterior limbs of the rats, the surgical posterior limbs of the rats occasionally showed slight claudication during walking, indicating that the injured nerve recovered well (Figure 2A). Rats in all groups were able to stand in balance, indicating that the innervation function of the injured nerve on the legs and feet of rats recovered well (Figure 2B). Compared with the normal sides of the rat, the paws of the surgical side of the rats in each group were not fully expanded, indicating that the re-innervation ability of the regenerated nerve had not recovered to the normal levels. In Autograft and BMSC-(BDNF + GDNF) groups, the degrees of expansion of the paws on the surgical posterior limbs were greater than those in control, BMSC-vector, BMSC-BDNF and BMSC-GDNF groups, indicating that the abilities of the regenerated nerve in Autograft and BMSC-(BDNF + GDNF) groups to reinnervate the rat feet were stronger than those in control, BMSC-vector, BMSC-BDNF and BMSC-GDNF groups.

### Neurologic Function Recovery

**FIGURE 3:**
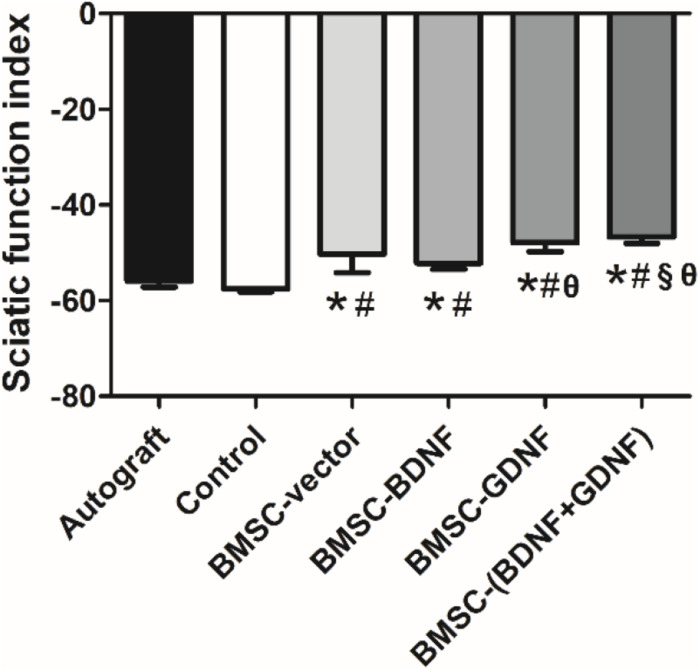
Analysis of SFI values in each group. *P < 0.05, compared with Autograft group; ^#^*P* < 0.05, compared with control group; ^§^*P* < 0.05, compared with BMSC-vector group; ^θ^*P* < 0.05, compared with BMSC-BDNF group.

Motor functional recovery in all groups was determined at 3 months after surgery (Figure 3). The mean sciatic function index (SFI) values of Autograft, control, BMSC-vector, BMSC-BDNF, BMSC-GDNF and BMSC-(BDNF + GDNF) groups were −55.89, −57.59, −50.27, −52.15, −47.88, and −46.68, respectively. The rats in BMSC-(BDNF + GDNF) group had better functional recovery, showing a higher SFI value than that in Autograft, control, BMSC-vector and BMSC-BDNF groups, but not higher than that in BMSC-GDNF group. The mean SFI value of BMSC-GDNF group was higher than those of Autograft, control and BMSC-BDNF groups, while the mean SFI value of BMSC-GDNF group was similar with that of BMSC-vector group.

### Neuroelectrophysiological Examination

**FIGURE 4:**
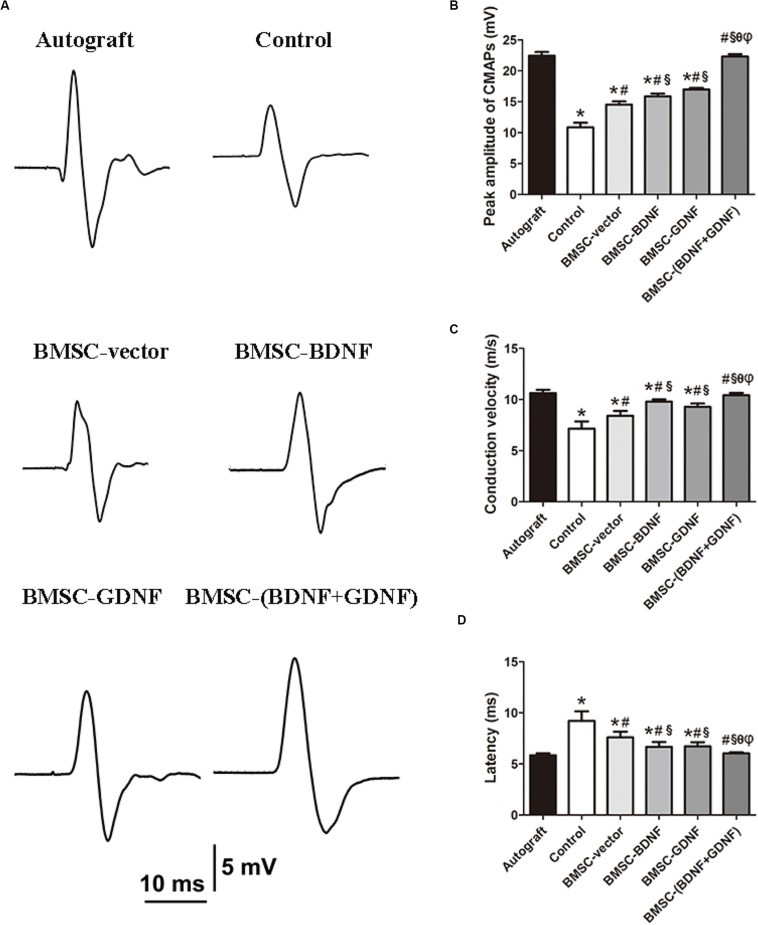
The results of electrophysiological test. **(A)** Representative CMAPs recordings on the injured side for each group. The statistical results of peak amplitude of CMAPs **(B)**, conduction velocity **(C)**, and latency **(D)**. *P < 0.05, compared with Autograft; ^#^*P* < 0.05, compared with control group; ^§^*P* < 0.05, compared with BMSC-vector group; ^θ^*P* < 0.05, compared with BMSC-BDNF group; ^φ^*P* < 0.05, compared with BMSC-GDNF group.

Three months after surgery, the peak amplitude of CMAPs, conduction velocity and latency of CMAPs were recorded by the biological signal acquisition and analysis system (Figure 4). Representative CMAPs record of the surgical side of each group are shown in Figure 4A. CMAPs signals could be detected in all groups, but the waveforms of signal were different among groups, indicating that the conduction functions of the damaged nerves were restored with different levels. The mean peak amplitude of CMAPs in Autograft, control, BMSC-vector, BMSC-BDNF, BMSC-GDNF, and BMSC-(BDNF + GDNF) groups were 22.44, 10.86, 14.51, 15.86, 16.98, and 22.31 mV, respectively. The peak amplitude of CMAPs in control, BMSC-vector, BMSC-BDNF and BMSC-GDNF groups were significantly lower than that in Autograft and BMSC-(BDNF + GDNF) groups, while there was no significant difference between BMSC-(BDNF + GDNF) group and Autograft group (Figure 4B). The peak amplitude of CMAPs in BMSC-BDNF and BMSC-GDNF groups were significantly higher than that in control and BMSC-vector groups, while the peak amplitude of CMAPs in BMSC-BDNF group was similar with that of BMSC-GDNF group. Compared with the peak amplitude of CMAPs in control, the peak amplitude of CMAPs in BMSC-vector was higher. These data indicated that the signal intensity of regenerative nerve in the BMSC-(BDNF + GDNF) group was closed to that of the Autograft group, while signal intensities in the BMSC-BDNF and BMSC-GDNF groups were higher than that of control and BMSC-vector groups.

According to Figures 4C,D, the conduction velocity of CMAPs in BMSC-(BDNF+GDNF) and Autograft groups were significantly higher than that in the control, BMSC-vector, BMSC-BDNF and BMSC-GDNF groups. The latency of CMAPs in BMSC-(BDNF + GDNF) and Autograft groups was significantly lower than that in control, BMSC-vector, BMSC-BDNF and BMSC-GDNF groups. The conduction velocity of CMAPs in BMSC-BDNF and BMSC-GDNF groups were higher than that in control and BMSC-vector groups, while that in BMSC-vector group was higher than that in control group. The latency of CMAPs in BMSC-BDNF and BMSC-GDNF groups were lower than that in control and BMSC-vector groups, while in BMSC-vector group was lower than that in control group. These data indicated that the electrical signal transmission speed of the regenerative nerve in BMSC-(BDNF+GDNF) group was higher than that in the control, BMSC-vector, BMSC-BDNF and BMSC-GDNF groups, while closed to that in the Autograft group. In conclusion, nerves repaired with HO-PSNCs conduits loaded with BDNF and GDNF co-transfected BMSCs had better recovery of conduction function and could transmit electrical signals faster than BDNF or GDNF single-transfected BMSCs, and the ability was similar to that of the Autograft group.

### HE Staining and Immunofluorescence Staining of Regenerated Nerves

**FIGURE 5:**
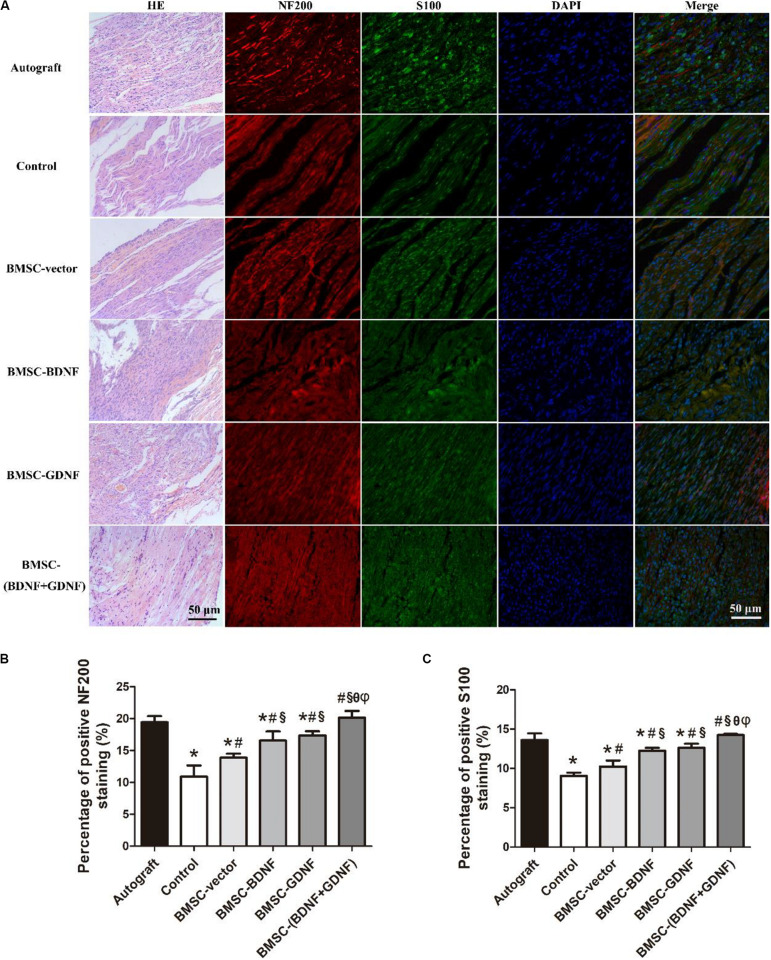
HE-staining and immunofluorescence analysis of the regenerated nerves in each group at 3 months after surgery. **(A)** Images of HE-staining and immunofluorescence; **(B)** Percentage of positive NF200 staining and **(C)** percentage of positive S100 staining. *P < 0.05, compared with Autograft; ^#^*P* < 0.05, compared with control group; ^§^*P* < 0.05, compared with BMSC-vector group; ^θ^*P* < 0.05, compared with BMSC-BDNF group; ^φ^*P* < 0.05, compared with BMSC-GDNF group.

Three months after surgery, the middle segments of the regenerated nerve were taken for HE staining, and the results are shown in Figure 5A. The regenerated nerve fibers in each group were arranged in order. These regenerated nerve fibers gathered into bundles, extending from the proximal end of the damaged nerve to the distal end. The regenerated nerve fibers in Autograft group were loose, while the regenerated nerve fibers in control, BMSC-vector, BMSC-BDNF, BMSC-GDNF, and BMSC-(BDNF + GDNF) groups were denser. The adhesion of the fiber bundles in control group was more dispersive than other conduit groups. In addition, the widths of the regenerated nerve tissues in the Autograft and BMSC-(BDNF+GDNF) groups were bigger than that of control, BMSC-vector, BMSC-BDNF and BMSC-GDNF groups.

The middle segments of the regenerated nerve were also taken for immunofluorescence staining of NF200 and S100, which NF200 is a specific marker of axon and S100 is the specific marker of Schwann cells, and the staining results are shown in Figure 5A. The Schwann cells and axons distribution of regenerated nerves were observed in all groups, indicating that the new regenerated tissue is indeed nerve tissue. The statistical results of positive cell percentages of NF200 and S100 are shown in Figures 5B,C. In Figure 5B, the positive cell percentage of NF200 in Autograft and BMSC-(BDNF + GDNF) groups were higher than that of control, BMSC-vector, BMSC-BDNF and BMSC-GDNF groups, while there was no difference between Autograft group and BMSC-(BDNF+GDNF) group. The positive cell percentage of NF200 in BMSC-BDNF and BMSC-GDNF groups were higher than that of control and BMSC-vector groups. Besides, the positive cell percentage of NF200 in BMSC-vector group was higher than that of control group. According to the Figure 5C, the positive cell percentage of S100 in Autograft and BMSC-(BDNF + GDNF) groups were higher than that of other groups, while the positive cell percentage of S100 in BMSC-BDNF and BMSC-GDNF groups were higher than that of control and BMSC-vector groups. In addition, the positive cell percentage of S100 in the BMSC-vector group was higher than that of control group.

In summary, these results showed that the nerve repair effect of BMSC-(BDNF + GDNF) group was highest among the conduit groups. The expression of NF200 and S100 of BMSC-vector group was higher than that of control group, while the expression of NF200 and S100 of BMSC-BDNF and BMSC-GDNF groups were higher than that of control and BMSC-vector groups.

### Toluidine Blue Staining and Transmission Electron Microscopy of Regenerated Nerve

**FIGURE 6:**
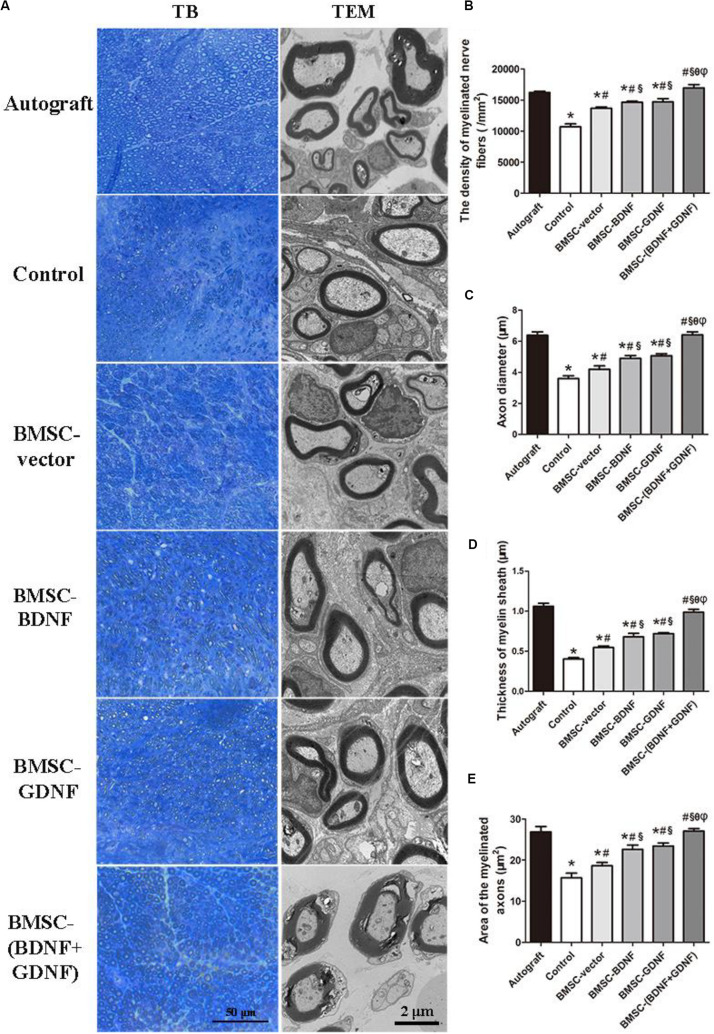
Toluidine blue (TB) staining and transmission electron microscopy (TEM) analysis of regenerated nerves. **(A)** Images of toluidine blue staining and TEM cross sections of regenerated nerve; **(B)** The statistical density of myelinated nerve fibers in regenerated nerves in each group; **(C)** Axon diameter, **(D)** thickness of myelin sheath and **(E)** area of the myelinated axon of regenerated nerves. *P < 0.05, compared with Autograft; ^#^*P* < 0.05, compared with control group; ^§^*P* < 0.05, compared with BMSC-vector group; ^θ^*P* < 0.05, compared with BMSC-BDNF group; ^φ^*P* < 0.05, compared with BMSC-GDNF group.

The cross sections of the middle segments of the regenerated nerve were further stained by toluidine blue (Figure 6A). The hollow myelinated nerve fibers could be clearly detected in each group, and these myelinated nerve fibers were evenly distributed in the regenerated nerve tissue. However, the density and size of myelinated nerve fibers were different in each group. The density of regenerated myelinated nerve fibers in Autograft, control, BMSC-vector, BMSC-BDNF, BMSC-GDNF, and BMSC-(BDNF + GDNF) groups were 16206.4, 10688.2, 13658.9, 14659.3, 14685.6, and 16940.5/mm^2^, respectively (Figure 6B). According to the statistical analysis, the density of myelinated nerve fibers in control, BMSC-vector, BMSC-BDNF, and BMSC-GDNF groups were significantly lower than that in Autograft and BMSC-(BDNF + GDNF) groups, while that in BMSC-BDNF and BMSC-GDNF groups was significantly higher than that in control and BMSC-vector groups. In addition, the density of myelinated nerve fibers in BMSC-vector group was significantly higher than that in control group. These data indicated that the formation of myelinated nerve fibers in BMSC-(BDNF + GDNF) group was higher than BMSC-BDNF and BMSC-GDNF groups, while the formation of myelinated nerve fibers in BMSC-BDNF and BMSC-GDNF group were higher than control and BMSC-vector groups.

Transmission electron microscopy (TEM) images of the cross-section of the middle portion of the regenerated nerves were shown in Figure 6A. The histomorphometric parameters of the regenerated nerves including axon diameter, myelin sheath thickness and area of the myelinated axons were investigated (Figures 6C–E) and based on Figure 6A. As shown in Figures 6A,C, the mean axon diameters in Autograft, control, BMSC-vector, BMSC-BDNF, BMSC-GDNF, and BMSC-(BDNF + GDNF) groups were 6.38, 3.6, 4.2, 4.9, 5.07, and 6.4 μm, respectively. The axon diameters in Autograft and BMSC-(BDNF + GDNF) groups were significantly larger than those in control, BMSC-BMSC, BMSC-BDNF, and BMSC-GDNF groups, while there was no difference in axon diameters between Autograft group and BMSC-(BDNF + GDNF) group. The axonal diameters in BMSC-BDNF and BMSC-GDNF groups were larger than those in control and BMSC-vector groups, and there was no difference of axon diameters between BMSC-BDNF group and BMSC-GDNF group. In addition, the axonal diameters in BMSC-vector group were larger than that of control group. As shown in Figures 6D,E, tendencies of thickness of myelin sheaths and area of the myelinated axons were consistent with that of the axon diameter. These results demonstrated that the myelin regeneration ability in BMSC-(BDNF + GDNF) group were better than those in control, BMSC-vector, BMSC-BDNF and BMSC-GDNF groups, which was similar to those in Autograft group. Furthermore, the myelin regeneration ability in BMSC-BDNF and BMSC-GDNF groups were better than that of control and BMSC-vector, while the myelin regeneration and re-innervation ability in BMSC-vector group was better than that of control group.

### Masson’s Staining of the Gastrocnemius Muscle

**FIGURE 7:**
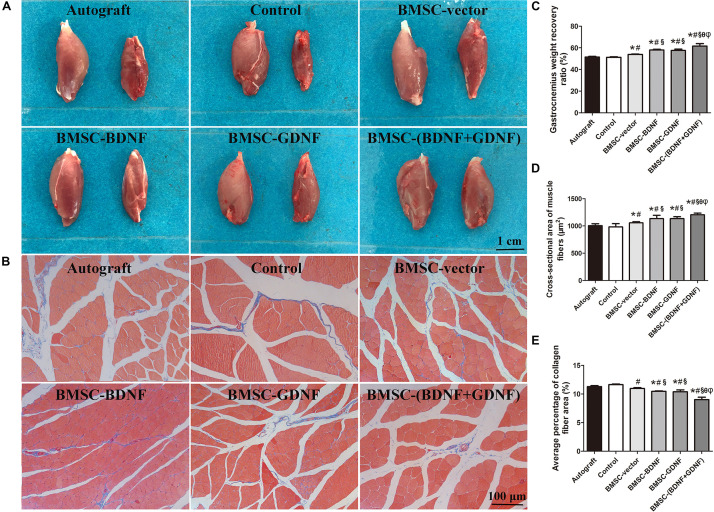
Gastrocnemius muscle analysis. **(A)** Images of gastrocnemius muscle between normal and operative sides; **(B)** Masson’s trichrome staining of cross sections of gastrocnemius muscle; **(C)** The gastrocnemius weight recovery ratio; **(D)** The cross sectional area of muscle fibers. **(E)** The average percentage of collagen fiber area. *P < 0.05, compared with Autograft; ^#^*P* < 0.05, compared with control group; ^§^*P* < 0.05, compared with BMSC-vector group; ^θ^*P* < 0.05, compared with BMSC-BDNF group; ^φ^*P* < 0.05, compared with BMSC-GDNF group.

The gastrocnemius muscles on the left and right sides of the rats were taken out and weighed 3 months after surgery. The gastrocnemius on the operative side in each group was smaller than that on the normal side (Figure 7A). As demonstrated in Figure 7C, the muscle weight recovery rates of Autograft, control, BMSC-vector, BMSC-BDNF and BMSC-GDNF groups were lower than that of BMSC-(BDNF + GDNF) group, while the muscle weight recovery rates of BMSC-BDNF and BMSC-GDNF groups were significantly higher than that of control and BMSC-vector groups. Moreover, the muscle weight recovery rate of BMSC-vector group was higher than that of control group. In the Masson’s staining images of the gastrocnemius muscle, the muscle fibers were stained red and the collagen fibers were stained blue (Figure 7B). Compared with BMSC-(BDNF + GDNF) group, the muscle fiber cross-sectional area of Autograft, control, BMSC-vector, BMSC-BDNF and BMSC-GDNF groups were significantly smaller, while the muscle fiber cross-sectional area of BMSC-BDNF and BMSC-GDNF groups were higher than that of Autograft, control and BMSC-vector groups (Figure 7D). Besides, the muscle fiber cross-sectional area of BMSC-vector group was higher than that of Autograft and control groups. As shown in Figure 7E, the collagen fiber area percentage of the BMSC-(BDNF + GDNF) group was significantly lower than that of Autograft, control, BMSC-vector, BMSC-BDNF and BMSC-GDNF groups, while the collagen fiber area percentage of BMSC-BDNF and BMSC-GDNF groups were significantly smaller than that of Autograft, control and BMSC-vector groups. Moreover, the collagen fiber area percentage of BMSC-vector group was smaller than that of Autograft and control groups. These results suggested that the re-innervation ability of the regenerated nerve in BMSC-BDNF and BMSC-GDNF groups were stronger than that of Autograft, control and BMSC-vector groups, while re-innervation ability of the regenerated nerve in BMSC-(BDNF + GDNF) group the was stronger than that of Autograft, control, BMSC-vector, BMSC-BDNF and BMSC-GDNF groups.

## Discussion

In this study, an efficient strategy for peripheral nerve repair is developed by combination of highly oriented nanofibrous nerve conduits (HO-PSNCs), seed cells (BMSCs), and neurotrophic factors (BDNF and GDNF). HO-PSNCs conduits could support peripheral nerve regeneration, but the repair effect was limited. The addition of seed cells (e.g., Schwann cells, Schwann cell-like cells and BMSCs) in nerve conduits were reported to promote nerve repair (Luo et al., 2015; Xue et al., 2017; Fu et al., 2019). Here, naïve BMSCs in HO-PSNCs conduits did improve the motor function, conduction function, nerve fiber morphology, protein expression, myelin regeneration and re-innervation ability of the regenerated nerve. However, other indexes of nerve regeneration had no significant change by naïve BMSCs. These data were consistent with the reports that BMSCs could promote conduit mediated nerve repair (Nijhuis et al., 2013; Gao et al., 2016).

Fu et al. (2011) reported that GDNF or BDNF transfected neural stem cells could promote the sciatic nerve regeneration, but he did not study the synergy effects in nerve regeneration. Although electrical stimulation can simultaneously promote the secretion of nutritional factors such as BDNF and GDNF during nerve regeneration, it has not been compared with single factor group (Willand et al., 2016). Wang et al. (2016) revealed that BDNF and GDNF fused to a laminin-binding domain in collagen tube had synergistical repair promoting effects of laryngeal nerve. Hoyng et al. (2014) identified the promoting effect of single factor BDNF, GDNF or NGF on the nerve regeneration, while the Hsu et al. constructed a CRISPR-based system for activating endogenous BDNF, GDNF, and NGF genes in adipose stem cell sheets to stimulate peripheral nerve regeneration (Hsu et al., 2019). In summary, no one has reported the synergistic effect of endogenous BDNF and GDNF (ratio = 1:1) on nerve regeneration.

Among the large number of neurotrophic factors, BDNF can promote the myelin formation of neogenesis nerves and GDNF can protect motor neurons from injury caused by nerve transection, which have been confirmed the role in the repair of PNI (Xiong et al., 2016; Lu et al., 2017). In our study, BMSCs overexpressing single factor exhibited many differences with naïve BMSCs group with improved conduction function, myelin regeneration and re-innervation ability of the regenerated nerve. All other indexes of nerve regeneration, such as SFI values and HE staining, had no significant change by introducing single neurotrophic factor to BMSC comparing to naïve BMSCs. These data were in constancy with the report that BMSC-BDNF need to combine with other factor to promote spinal cord recovery (Xiong et al., 2016). Altogether, naïve BMSCs, BMSCs overexpressing BDNF and BMSCs overexpressing GDNF as seed cells could efficiently improve peripheral nerve regeneration.

Interestingly, mixing BMSCs overexpressing BDNF and BMSCs overexpressing GDNF as seed cells in our study greatly elevated the repair of peripheral nerves. All BMSCs groups included the same cell number (5 × 10^4^ cells). In this case, the expression levels of BDNF or GDNF in the double-factor group was half as much as that in the single-factor group. However, all the indexes of nerve regeneration were better than those of the single-factor groups. These data suggest that not the higher dose of single neurotrophic factors would make the better the effect of nerve regeneration, but BDNF and GDNF could synergistically promote nerve regeneration more effectively with different mechanisms of action. BDNF secreted by BMSCs can bind to p75NTR receptors of Schwann cells to promote myelinization (Xiao et al., 2009). GDNF has a strong role in promoting survival and growth of motor neurons, and the sciatic nerve contains motor and sensory axons emitted from spinal cord and DRG cells (Eric et al., 2002). The two factors functional in different aspects of nerve repair may collaborate in our study making high efficiency. Although the ratio of GDNF producing BMSC and BDNF producing BMSC need to be further optimized, our study provides an excellent system of mixing different seed cells with synergistic ability for nerve repair.

## Conclusion

BMSCs overexpressing BDNF and BMSCs overexpressing GDNF were constructed and combined with the HO-PSNCs for peripheral nerve repair. Mixture of BMSCs overexpressing BDNF and BMSCs overexpressing GDNF as seed cells greatly improved the sciatic nerve repair comparing to the BMSCs-BDNF or BMSCs-GDNF single factor groups and similar to autograft group. Therefore, our study not only provided an optimal method of stem cell-based, multiple factors-mediated and conduit-guided for nerve repair, but also came up with a new strategy of seed cell mixture with synergistic ability for nerve repair.

## Data Availability Statement

The raw data supporting the conclusions of this article will be made available by the authors, without undue reservation.

## Ethics Statement

The animal study was reviewed and approved by Animal Care and Use Committees of Wuhan University.

## Author Contributions

QZ, PW, and YC conceived the initial idea and designed the experiments. QZ, PW, FC, and YZ performed the experiments. QZ, PW, and ZT analyzed the data and wrote the manuscript. YL, XH, CH, QY, ZT, and YC revised the manuscript. YC and ZT worked on funding acquisition. All the authors have read and approved the final version of the manuscript.

## Conflict of Interest

QZ was employed by the company Hangzhou Singclean Medical Products Co., Ltd. The remaining authors declare that the research was conducted in the absence of any commercial or financial relationships that could be construed as a potential conflict of interest.
