# Supplementary material for: Brain Derived Neurotrophic Factor and Glial Cell Line-Derived Neurotrophic Factor-Transfected Bone Mesenchymal Stem Cells for the Repair of Periphery Nerve Injury
Source: Front Bioeng Biotechnol. 2020 Jul 30;8:874. doi: 10.3389/fbioe.2020.00874 (PMC7406647; doi:10.3389/fbioe.2020.00874)
Supplement: Supplementary file 1 [file Data_Sheet_1.PDF]

Brain derived neurotrophic factor and glial cell line-derived neurotrophic factor-transfected bone mesenchymal stem cells for the repair of periphery nerve injury

Qiang Zhang[1, 2#], Ping Wu[1#], Feixiang Chen[1], Yanan Zhao[1], Yinping Li[1], Xiaohua He[1], Céline Huselstein[3], Qifa Ye[4, 5], Zan Tong[1*] and Yun Chen[1, 5*]

1 Department of Biomedical Engineering and Hubei Province Key Laboratory of Allergy and Immune Related Diseases, School of Basic Medical Sciences, Wuhan University, Wuhan 430071, China

2 Hangzhou Singclean Medical Products Co., Ltd., Hangzhou 310018, China

3 CNRS UMR 7561 and FR CNRS-INSERM 32.09 Nancy University, Vandœuvre-lès-Nancy, France

4 Zhongnan Hospital of Wuhan University, Institute of Hepatobiliary Diseases of Wuhan University, Transplant Center of Wuhan University, Wuhan 430071, China

5 Hubei Engineering Center of Natural Polymers-based Medical Materials, Wuhan University, Wuhan 430071, China

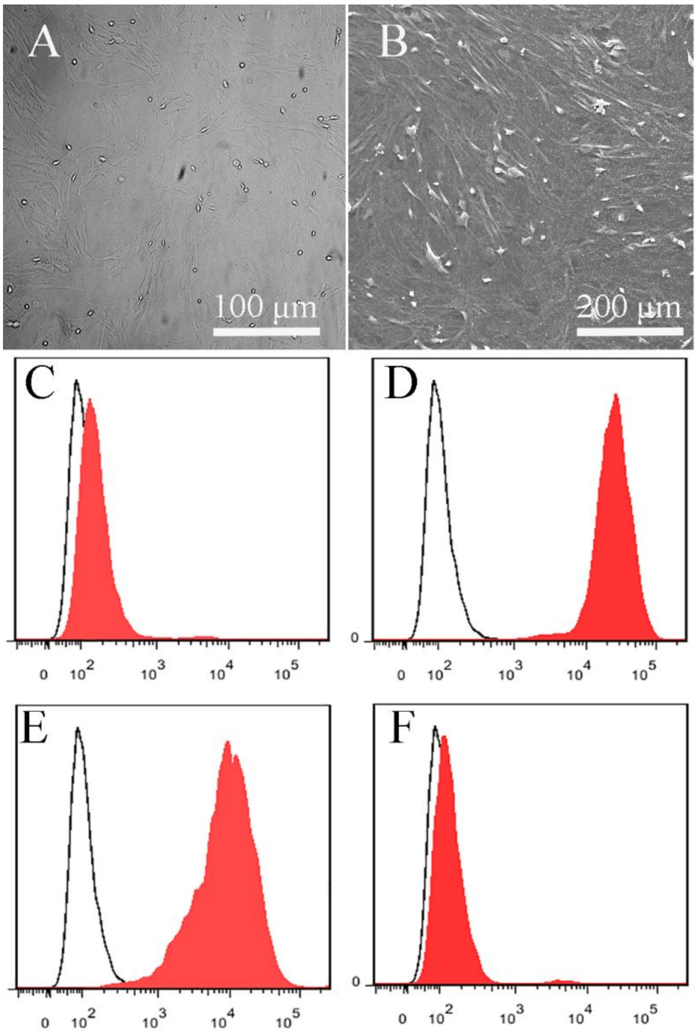

Figure S1. Characterization of BMSCs. (A) Optical images and (B) SEM images of BMSCs. (C) CD11b, (D) CD29, (E) CD90 and (F) CD45 expressions of BMSCs checked by the flow cytometry.
